# Transpancreatic biliary sphincterotomy using a novel rotatable sphincterotome in a patient with Roux-en-Y gastrectomy

**DOI:** 10.1055/a-2603-5448

**Published:** 2025-06-03

**Authors:** Yuki Tanisaka, Shomei Ryozawa, Masafumi Mizuide, Akashi Fujita, Ryuichi Watanabe, Kengo Komori

**Affiliations:** 1 183786 Department of Gastroenterology, Saitama Medical University International Medical Center, Hidaka, Japan; 2 Department of Gastroenterology, Saitama Medical University International Medical Center, Hidaka, Japan

Biliary cannulation in patients with Roux-en-Y gastrectomy under balloon enteroscopy is challenging 1 2 . When pancreatic duct cannulation is performed, transpancreatic biliary sphincterotomy is a useful rescue technique for achieving biliary cannulation in difficult cases. However, transpancreatic biliary sphincterotomy in patients with Roux-en-Y gastrectomy is considered difficult as the blade of conventional sphincterotomes does not always face the correct direction for the incision. Recently, a novel rotatable sphincterotome (ENGETSU; Kaneka Corp., Osaka, Japan) was launched, which allows the blade to be rotated in the correct direction for endoscopic sphincterotomy by turning the handle ( Fig. 1 ) 3 4 . We report a case of Roux-en-Y gastrectomy in which transpancreatic biliary sphincterotomy was successfully performed using a novel rotatable sphincterotome to achieve selective biliary cannulation.

**Fig. 1:**
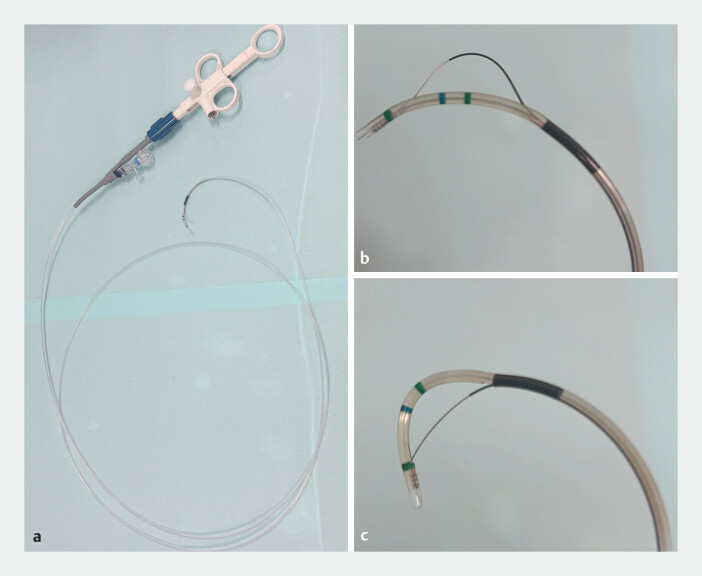
The novel rotatable sphincterotome (ENGETSU; Kaneka, Japan). **a** The product overview. **b** The blade can be loosened. **c** The blade can be also stretched.

A 79-year-old man who underwent Roux-en-Y gastrectomy and presented with a biliary stricture caused by the recurrence of gastric cancer was referred to us ( Fig. 2 ). Endoscopic retrograde cholangiopancreatography was performed using a short-type single-balloon enteroscope (SIF-H290; Olympus, Tokyo, Japan) with a working length of 152 cm and a working channel 3.2 mm in diameter 5 ( Video 1 ). Since only pancreatic duct cannulation was successful, the double-guidewire technique was used; however, selective biliary cannulation remained difficult to achieve. ( Fig. 3 **a–c** ). Subsequently, transpancreatic biliary sphincterotomy was attempted using a novel rotatable sphincterotome. Although the blade of the sphincterotome was initially not facing the 5 oʼclock direction ( Fig. 4 **a** ), which corresponds to the bile duct, it was adjusted to face 5 oʼclock by turning the handle, and a successful incision was made ( Fig. 4 **b, c** ). The orifice of the bile duct was identified at the incision site ( Fig. 4 **d** ), allowing selective biliary cannulation to be achieved ( Fig. 4 **e, f** ). Finally, biliary drainage was completed ( Fig. 5 ).

**Fig. 2:**
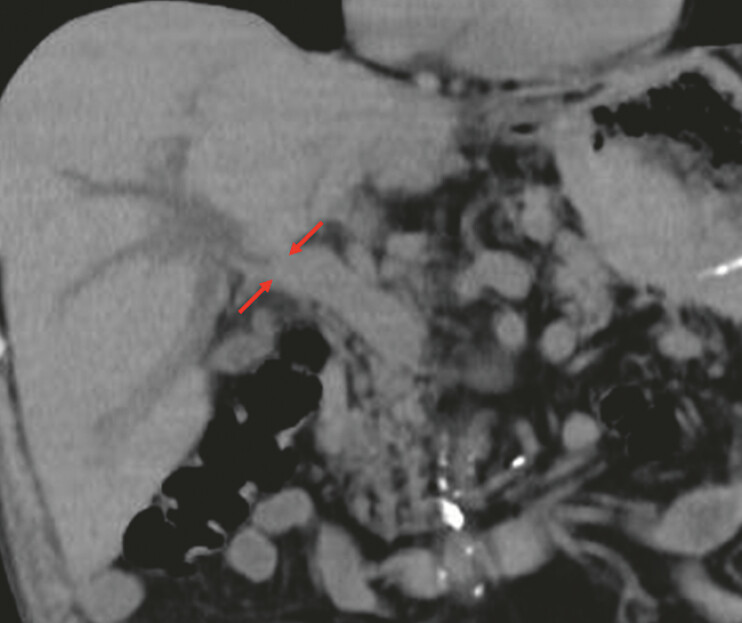
Computed tomography revealing biliary stricture (red arrow) in the hilar bile duct.

Successful transpancreatic biliary sphincterotomy using a novel rotatable sphincterotome in a patient with Roux-en-Y gastrectomy.Video 1

**Fig. 3:**
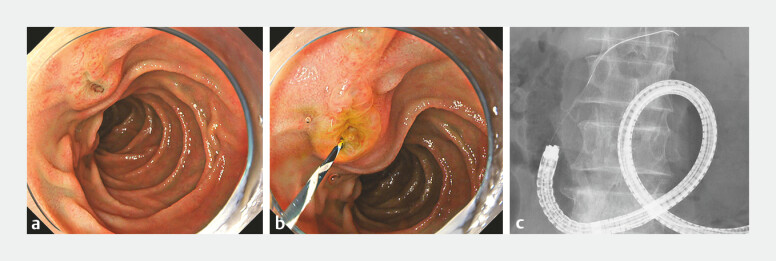
Endoscopic retrograde cholangiopancreatography findings. **a** The finding of the papilla. **b, c** Selective biliary cannulation is attempted; however, only pancreatic duct cannulation is successful, despite the use of the double-guidewire technique.

**Fig. 4:**
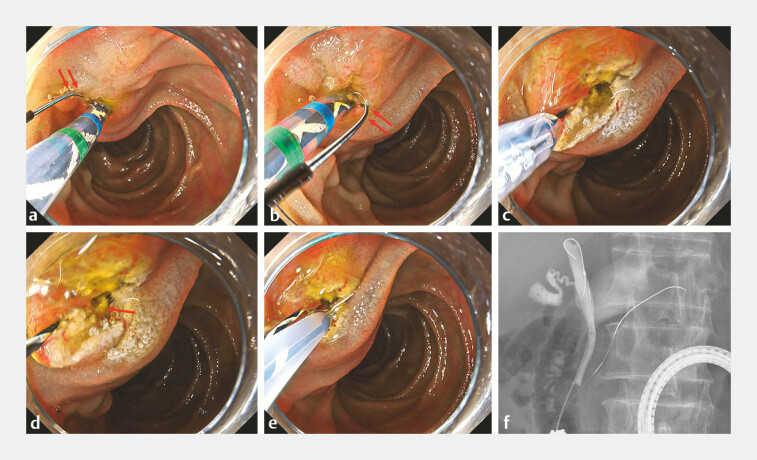
Endoscopic retrograde cholangiopancreatography findings. **a** The blade of the sphincterotome was initially not facing the 5 oʼclock direction (red arrow). **b** It is adjusted to face the 5 oʼclock direction (red arrow) by turning the handle. **c** A successful incision is made. **d** The bile duct orifice is identified at the incision site (red arrow). **e, f** Selective biliary cannulation is achieved.

**Fig. 5:**
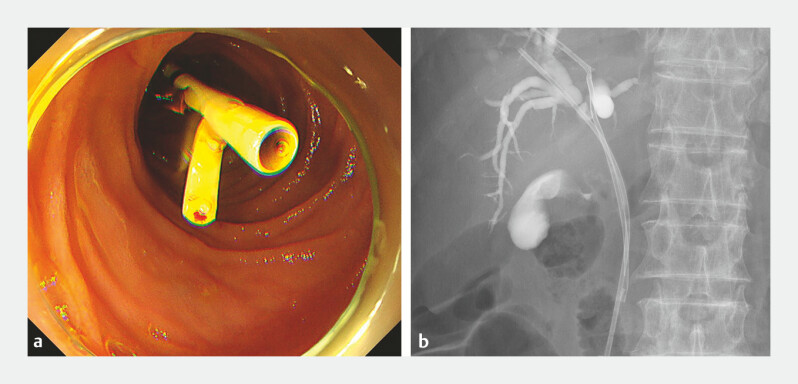
Endoscopic retrograde cholangiopancreatography findings. **a,b** Finally, biliary drainage is completed.

This novel rotatable sphincterotome could improve advanced selective biliary cannulation techniques such as transpancreatic biliary sphincterotomy under balloon enteroscopy.

Endoscopy_UCTN_Code_TTT_1AR_2AC

## Notes

Endoscopy E-Videos https://eref.thieme.de/e-videos
*E-Videos* is an open access online section of the journal *Endoscopy* , reporting on interesting cases and new techniques in gastroenterological endoscopy. All papers include a high-quality video and are published with a Creative Commons CC-BY license. Endoscopy E-Videos qualify for HINARI discounts and waivers and eligibility is automatically checked during the submission process. We grant 100% waivers to articles whose corresponding authors are based in Group A countries and 50% waivers to those who are based in Group B countries as classified by Research4Life (see: https://www.research4life.org/access/eligibility/ ). This section has its own submission website at https://mc.manuscriptcentral.com/e-videos .
